# Difference in Therapeutic Strategies for Joint‐Preserving Surgery for Non‐Traumatic Osteonecrosis of the Femoral Head between the United States and Japan: A Review of the Literature

**DOI:** 10.1111/os.12979

**Published:** 2021-04-05

**Authors:** Yutaka Kuroda, Yaichiro Okuzu, Toshiyuki Kawai, Koji Goto, Shuichi Matsuda

**Affiliations:** ^1^ Department of Orthopaedic Surgery Graduate School of Medicine, Kyoto University Kyoto Japan

**Keywords:** Japan, Joint‐preserving surgery, Osteonecrosis, Review, Total hip arthroplasty

## Abstract

For patients with non‐traumatic osteonecrosis of the femoral head (ONFH), core decompression (CD) and bone grafts (BG) are mainly performed in the West, while osteotomy is found to be predominant in Japan. It is not well recognized how the surgical procedures for joint preservation in patients with ONFH are completely different between the United States and Japan. This paper identifies the contexts and the differences in treatment strategies for ONFH between the two countries. We compared the surgical trends of the two countries over three periods, 1997–2001, 2002–2006, and 2007–2011 (the US data for the third period was 2007–2008), based on a 2014 US paper and a 2013 national publication in Japan. We compared the details of surgery for non‐traumatic ONFH under the same conditions in the two reports. For the period 1997–2001, the rates of surgeries for ONFH in the US were as follows: total hip arthroplasty (THA), 86%; CD, 10%; and osteotomy, 0.4%. In Japan, THA was 61%, osteotomy 38%, and CD 0%. For the recent period, 2007–2011 (US 2007–2008), the rate of THA was 91%, CD 6%, and osteotomy 0.1%, in the US, compared to a THA rate of 73%, CD 0%, and osteotomy 26% in Japan. The results for the interim period (2002–2006) were between the old and new data. The use of joint‐preserving surgery for ONFH differs greatly between the US and Japan. The first‐line joint‐preserving surgery was CD in the US and osteotomy in Japan. Each procedure was rarely done in the other country. From about 2000 to 2010, the percentage of THA increased in both countries. The proportion of joint‐preserving surgery (CD in the US and osteotomy in Japan) declined. The decrease in joint‐preserving procedures may be largely attributed to improved long‐term outcomes of THA due to technological advances. There is also a reluctance for young ONFH patients to undergo joint‐preserving procedures, such as osteotomy, that require long‐term hospitalization.

## Graphical abstract

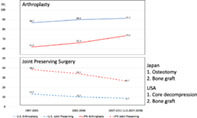

A comparison of the ONFH procedures between 2000 and 2010 in the US and Japan showed that the use of joint‐preserving techniques was very different. First‐line joint‐preserving surgery is CD in the US, while it is osteotomy in Japan. Each country rarely performed the alternative technique.

## Introduction

Osteonecrosis of the femoral head (ONFH) has been identified as an incurable disease of the hip joint that occurs in young patients. The etiology of non‐traumatic ONFH is multifactorial. Although it is not completely understood, systemic corticosteroid pulse therapy and heavy alcohol consumption are considered to be the major factors in the development of non‐traumatic ONFH^1^, ^2^, ^3^. It occurs at around 40 years of age and is the main reason for total hip arthroplasty (THA) in young patients. For patients with ONFH, the femoral head collapse is significantly related to impact on activity of daily living. Therefore, the primary concern for the surgeon and the patients is joint preservation, and avoiding the collapse of the femoral head. Recently, corticosteroid‐associated ONFH has been increasingly recognized and can be diagnosed at an early stage with careful evaluation using magnetic resonance imaging (MRI)^4^. Collapse of the femoral head is more likely when the extent of osteonecrosis is great. In our previous study of 505 ONFH hips^5^, 85% of ONFH patients with the most extensive disease developed radiological collapse at 5 years. Additionally, half of the patients had already progressed to collapse at the time of initial diagnosis, and more than 60% of the cases were found to have bilateral ONFH.

THA has been considered as the final treatment option for end‐stage osteoarthritis (OA) secondary to femoral head collapse. Many good results for THA in ONFH have now been reported, which are comparable to those of THA in OA^6^, ^7^. It has been agreed that the optimal treatment for final‐stage ONFH and end‐stage OA is THA. There is, however, no consensus on the strategy for joint‐preserving surgery in the current guidelines and summaries for ONFH that have been published in different countries^1^, ^2^, ^3^, ^6^, ^7^. Historically, core decompression (CD) and bone grafts (BG) were performed to preserve joints in the West, while osteotomy was developed and used in Japan and became the mainstream^8^, ^9^, ^10^, ^11^, ^12^. ONFH, as an intractable disease, has long been a Japanese government research project from 1975. Some Japanese physicians have been aware of the differences in the surgical procedures between the West and Japan; however, until now, these differences have not been clarified. There are many guidelines available^1^, ^2^, ^3^, but there are only few reports with actual numbers and rates of surgery for ONFH. Our research uncovered only one article from the United States and the national publication from Japan (written in Japanese) that reported the numbers and ratios of surgical procedures for ONFH.

Recently, regenerative therapies with CD, including cell therapy^13^, ^14^, alternative bone therapy^15^, and growth factor therapy^16^, have been developed and are gaining in popularity as the next generation of treatments. With the advent of regenerative medicine, joint‐preserving treatment for ONFH is now facing a major crossroads. It is important to understand the differences in current treatment strategies used between the US and Japan when deciding on the ideal therapeutic strategies to overcome this intractable disease.

The purpose of the present study is: (i) to clarify the difference in therapeutic strategies for joint‐preserving surgery for non‐traumatic ONFH between the US and Japan; (ii) to collect the data based on a 2014 report from the US^17^ and data published in 2013 by the Japanese ONFH research group^18^; and (iii) to report the surgical procedures for ONFH in the three periods for the US and Japan.

## Methods

### The Comparative Study of Two Reports from the US and Japan

The US data on the surgical procedures for non‐traumatic ONFH were retrieved from an article authored by Johnson *et al*.^17^. Meanwhile, the Japanese data were retrieved from the published report on non‐traumatic ONFH in 2013 as part of the Ministry of Health, Labour and Welfare's intractable disease project in Japan (Yukihide Iwamoto, written in Japanese)^18^.

### The US Data

The US article sought to determine the trends in the types and numbers of procedures performed for secondary, non‐traumatic ONFH from 1992 to 2008 in the US, based on data from the National Inpatient Sample (NIS). The NIS is a database maintained by the Agency for Healthcare Research and Quality. It contains a longitudinally representative sample of 20% of the hospitals in the US and tracks approximately 8 million hospitalizations annually. In 2013, the NIS was able to collect data from 1051 hospitals in 45 states. This was a 20% stratified sample of all hospitals in the US; using sampling weights, a statistically valid national total could be estimated. All patients who had an International Classification of Diseases, 9th Revision, diagnosis of ONFH between 1992 and 2008 were evaluated. The analysis was performed by extracting the following procedure codes from the NIS database: CD, nonvascular BG, free vascularized fibular graft, angular or rotational osteotomy, limited femoral head resurfacing, THA, conversion to THA, and revision THA.

### The Japanese Data

The Japanese 2013 report was an annual summary by the ONFH working group, written in Japanese. It included data on patients with non‐traumatic ONFH who underwent surgery at 30 high‐volume hospitals across the country over a 15‐year period from January 1997 to December 2011. The study included 3103 hips in 2430 patients. The main research items were surgical procedure, age at definitive diagnosis, background factors for developing ONFH, and the preoperative ONFH type and stage both based on the Japanese Investigation Committee (JIC) classification. The surgical procedures were categorized as follows: osteotomy, BG, bipolar hip arthroplasty (BHA), THA, revision BHA, revision THA, and others, including CD, hip resurfacing, or removal of implants. Questionnaires containing these items were mailed to the data center for tabulation and analysis. The 15‐year period was divided into three 5‐year periods (1997–2001, 2002–2006, and 2007–2011), for which we examined the trends of surgical procedures.

### Comparisons in Surgical Procedures for ONFH in the Three Periods for the US and Japan

We referred to the number of surgeries in 1992 and 2008, the years for which real numbers were provided in the US data. The total number of surgeries in Japan shown for each of the three time periods was referenced. The common study period for both reports was 1997–2008. The data for the US were reanalyzed in three 5‐year datasets: 1997–2001, 2002–2006, and 2007–2011. Since the US data ended in 2008, the most recent dataset compares the 2007–2008 period for the US to the 2007–2011 period for Japan. Since the US data were presented in a graph with no actual values shown, the graph was enlarged and used to extract the data to one decimal place using digital graph paper. The percentages of each operation for all primary surgery were calculated.

### Classification of Surgical Procedures for ONFH

For comparison, surgical techniques were unified as follows: for the US data, nonvascular BG and free vascularized fibular graft were combined as BG and conversion THA and revision THA were combined and designated as revision THA. Revision surgeries, including revision THA, revision BHA, and conversion to THA, were excluded because they were not primary surgeries for ONFH. Since CD and hip resurfacing (HR) were also not often performed, the numbers of CD and HR in Japan were set at 0. Most of the “others” classified in the Japanese data were the removal of metal implants after osteotomy. We excluded the “others” classified in the original Japanese data from the analysis of total surgical procedures. The original US report included limited femoral head resurfacing (synonymous with HR) as a “joint‐preserving” procedure, whereas in the present study, limited femoral head resurfacing was categorized in “arthroplasty” procedures. In summary, this comparative study analyzed the percentages of surgical procedures for primary surgery in patients with ONFH, separating “joint‐preserving” and “arthroplasty,” aligned with the conditions of the data reported in the two countries.

### Ethical Statement

This paper is a retrospective review article summarizing two publications written in different languages. Therefore, no protocol or relevant ethics committee approval was required.

## Results

### Changes in the Total Number of Surgical Procedures for ONFH

The US data representing a 20% sample of hospitals in the US showed that there were 3570 surgeries for ONFH in 1992 and 6400 surgeries in 2008. Data from 30 high‐volume institutions in Japan showed that primary operations for non‐traumatic ONFH totaled 640 in the period 1997–2001, 757 from 2002–2006, and 1417 from 2007–2011.

### Changes in Surgical Procedures for ONFH in the Three Periods for the US and Japan

**Fig 1:**
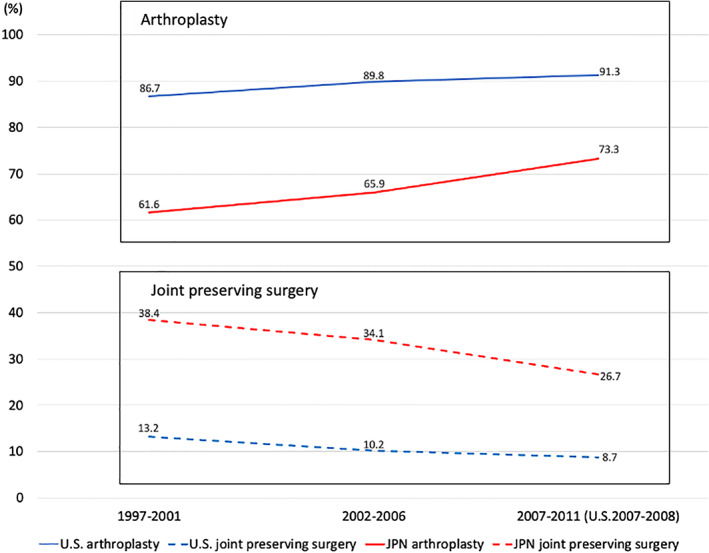
Changes in the percentages of arthroplasty and joint‐preserving surgery in the United States and Japan. The category of “arthroplasty” includes total hip arthroplasty, bipolar hip arthroplasty, and hip resurfacing (HR). In the original US data, HR was included in “joint preserving.” However, in this study, it was reanalyzed as arthroplasty (shown in Tables 1, 2, 3). Abbreviations: US, the United States; JPN, Japan.

**Fig 2:**
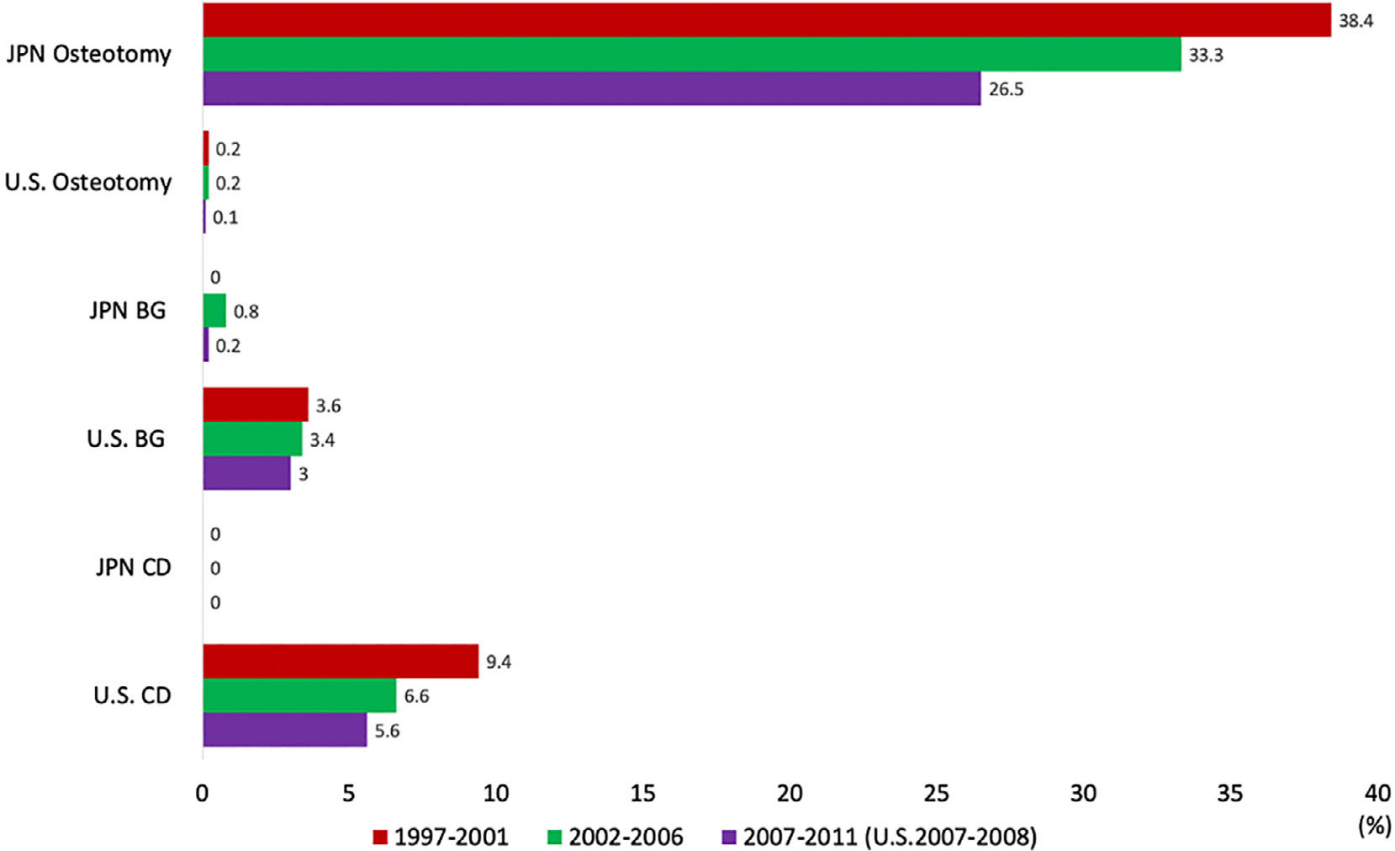
Comparison of joint‐preserving surgery over three periods in the United States and Japan. Bone graft (BG) includes both vascularized and free bone grafting. There are no similarities between the United States and Japan in terms of surgical techniques. The core decompression (CD) was the most commonly performed joint‐preserving surgery in the United States, but it was hardly performed in Japan. On the contrary, osteotomies had been mainstreamed in Japan but were hardly performed in the United States. All of the joint preserving surgeries share a gradually decreasing percentage. Abbreviations: US, the United States; JPN, Japan, CD, core decompression; BG, bone graft.

Details of the percentage of surgical procedures for ONFH in each period are summarized in Tables 1, 2, 3. Changes in the arthroplasty and joint‐preserving surgery rates in the US and Japan are shown in Fig. 1. A comparison of joint‐preserving surgery for the three periods in both countries is shown in Fig. 2. HR was described in the original US paper as partial THA or limited femoral head resurfacing.

**TABLE 1:** Surgical procedure for osteonecrosis of the femoral head in 1997 to 2001

United States			Japan		
Period	Procedure	Rate (%)	Period	Procedure	Rate (%)
1997–2001	“Arthroplasty”	86.7	1997–2001	“Arthroplasty”	61.6
	‐Total hip arthroplasty	78.2		‐Total hip arthroplasty	42.8
	‐Hip resurfacing	8.5		‐Bipolar hip arthroplasty	18.7
	“Joint preserving”	13.2		“Joint preserving”	38.4
	‐Core decompression	9.4		‐Core decompression	0
	‐Non‐vascularized bone graft	3.2		‐Bone graft	0
	‐Vascularized bone graft	0.4			
	‐Osteotomy	0.2		‐Osteotomy	38.4

All values in the table represent the average percentage of primary surgical procedures for the patients with non‐traumatic osteonecrosis of the femoral head. The US data were traced from the published graphs to estimate the real value using digital graph paper. The Japanese data were reanalyzed by the actual number in the original report. In Japan, joint‐preserving procedures can be considered the equivalent of an osteotomy.

**TABLE 2:** Surgical procedure for osteonecrosis of the femoral head in 2002 to 2006

United States			Japan		
Period	Procedure	Rate (%)	Period	Procedure	Rate (%)
2002–2006	“Arthroplasty”	89.8	2002–2006	“Arthroplasty”	65.9
	‐Total hip arthroplasty	83.6		‐Total hip arthroplasty	46.4
	‐Hip resurfacing	6.2		‐Bipolar hip arthroplasty	19.5
	“Joint preserving”	10.2		“Joint preserving”	34.1
	‐Core decompression	6.6		‐Core decompression	0
	‐Non‐vascularized bone graft	2.7		‐Bone graft	0.8
	‐Vascularized bone graft	0.7			
	‐Osteotomy	0.2		‐Osteotomy	33.3

In the US, arthroplasty accounted for about 90%, and in Japan it was 66%. One‐third of the surgical patients with osteonecrosis of the femoral head in Japan had undergone osteotomy.

**Table 3:** Surgical procedure for osteonecrosis of the femoral head in 2007 to 2011

United States			Japan		
Period	Procedure	Rate (%)	Period	Procedure	Rate (%)
2007–2008	“Arthroplasty”	91.3	2007–2011	“Arthroplasty”	73.3
	‐Total hip arthroplasty	87.2		‐Total hip arthroplasty	61.9
	‐Hip resurfacing	4.1		‐Bipolar hip arthroplasty	11.4
	“Joint preserving”	8.7		“Joint preserving”	26.7
	‐Core decompression	5.6		‐Core decompression	0
	‐Non‐vascularized bone graft	2.4		‐Bone graft	0.2
	‐Vascularized bone graft	0.6			
	‐Osteotomy	0.1		‐Osteotomy	26.5

In the US, arthroplasty increased over 90%, and in Japan it increased over 70%. On the other hand, joint‐preserving surgery in Japan decreased.

**Table 4:** Characteristics of joint‐preserving surgery

Procedure	Year of the first report author	Rehabilitation period	Derivative surgical technique	Conversion to THA	Invasiveness
Bone graft	1949 Phemister^22^	Several weeks	1. Non‐vascularized bone graft ‐1a. Light bulb ‐1b. Trapdoor 2. Vascularized bone graft ‐ 2a. Fibular graft ‐2b. Iliac graft	Easy Easy or moderate Difficult: Stem insertion for the hard fibula Easy	Moderate High* High Moderate or high
Osteotomy	1971 Nishio^10^	Several months	1. Intertrochanteric curved osteotomies 2. Transtrochanteric rotational osteotomies	1, 2. Difficult: Removal of metal implants, stem selection for the angulated femur.	High High
Core decompression	1960s Ficat^20^	Several days	1. Tantalum rod 2. Bone substitute 3. Cell therapy, growth factor	Difficult: Removal of tantalum implants Easy Easy	Moderate Minimum Minimum

Conversion to total hip arthroplasty (THA) was determined by the level of difficulty from previous joint‐preserving surgery. “Easy” can be performed just like a normal primary THA. “Difficult” was assumed to be more difficult than normal primary THA. The reason for the difficulty is stated after the semicolon. Invasiveness classified joint‐preserving surgery into three degrees. “High” requires an osteotomy, surgical dislocation of the hip*, or revascularization with microscopy. “Minimum” is a percutaneous procedure; “Moderate” is between “High” and “Minimum”.

Abbreviation: THA, total hip arthroplasty.

### 1997–2001

In Japan, the percentage of BHA instead of HR is high, second to THA. CD and bone graft surgery account for <1% of the total in Japan. In the US, the baseline for arthroplasty was as high as 86%. In Japan, the baseline for joint preservation was as high as just under 40%.

### 2002–2006

In the US, the percentage of arthroplasty was as high as 90%. The proportion of arthroplasty in Japan was close to two‐thirds of the surgical patients with ONFH. In the US, CD declined from 9.4% to 6.6% in this period.

### 2007–2011

The most recent dataset compares the 2007–2008 period for the US to the 2007–2011 period for Japan. In the US, the percentage of arthroplasty has exceeded to 90%. In Japan, the decline of joint preservation has been prominent in this period. In the US, HR declined to 4.1%. In Japan, the percentage of BHA declined from 19.5% to 11.4% in this period.

## Discussion

### Summary of the Major Results of the Study

In this comparative study of two reports from the US and Japan, procedures for joint‐preserving surgery in ONFH were found to be markedly different. The first option for joint‐preserving surgery was CD in the US, whereas it was osteotomy in Japan, and neither option has been available in the other country. During 1997–2001, 2002–2006, and 2007–2011 (the US data used covered 2007–2008), there was an increase in the ratio of THA, which was common to both countries. The data from the US and Japan showed an increase in the total number of ONFH surgeries, but the total number of joint‐preserving surgeries did not increase as much as the number of surgeries. The results show that the two countries increased the number of THAs and experienced a decrease in the number of joint‐preserving procedures.

### The Possible Reasons for the Increase in Total Hip Arthroplasty

There are several possible reasons for the increase in THA. Until the 1990s, there had been several studies reporting poor outcomes for THA compared to those for OA. More recent studies, however, have reported improved results^6^, ^7^. The advent of surface bearings with lower wear rates has led to promising results when used in patients with advanced ONFH as well as patients with OA. The emergence of highly cross‐linked polyethylene, in particular, was able to improve the long‐term performance of THA, as shown by national registry data^19^. In the US, HR has been followed by THA but is on the decline due to metal‐on‐metal issues. In Japan, BHA has been performed, followed by THA, but it is also decreasing, possibly due to long‐term problems such as central migration of the outer cup.

### Core Decompression and Cell Therapy

A similar increase in the number of THAs was observed in both countries. However, there was a completely different therapeutic strategy for ONFH with regard to joint‐preserving surgery. In the US, CD had been the predominant procedure for joint‐preserving surgery. CD has a long history dating back to the 1960s^20^. At first, single 10 mm core diameters were used, but since 2000, multiple CDs, which are 3 mm or less in diameter, have been preferred. More recently, due to advances in regenerative medicine, cell therapy combined with the CD procedure has become popular^1^, ^2^, ^3^, ^4^, ^13^, ^14^, ^15^, ^16^. For cell therapy, bone marrow mononuclear cells are mostly used. Cultured bone marrow stem cells or platelet‐rich plasmas have also been tried^1^, ^2^, ^3^, ^4^, ^13^. In contrast, in Japan, CD has not been performed at all except for biopsy purposes. Recently, CD‐based cell therapy has become available at just one university hospital in Japan^21^.

### Osteotomy

For 50 years, osteotomy has been the preferred surgical option for joint preservation in Japan. Since CD and BG have rarely been performed, joint‐preserving surgery can be considered the equivalent of an osteotomy. The idea behind osteotomy is to move the necrotic area from the loading surface of the femoral head. The adjustment is triaxial, including varus‐valgus, flexion‐extension, and anterior–posterior rotation. There are two main types of proximal femoral osteotomy: transtrochanteric rotational osteotomy, the preferred procedure, and intertrochanteric curved osteotomy, both developed in Japan^10^, ^11^. These procedures are technically demanding and less used in other parts of the world^1^, ^2^, ^3^, ^4^, but they are found to be popular in Asia^8^, ^9^, ^12^. Compared to other procedures, osteotomy requires longer post‐operative rehabilitation^10^, ^11^, ^12^. Current reports of ONFH from the US point out that these osteotomies are difficult to perform, have variable results, and can only be used in a select group of patients with small lesions^2^. It has also been noted that if the osteotomy fails, the subsequent conversion to THA is more difficult.

### Bone Grafting

With regard to bone grafting, various techniques using autologous bone have been reported. In the 1940s, Phemister used non‐vascularized bone from the tibia for the treatment of ONFH^22^. After Phemister's procedure, newer techniques such as the light bulb and the trapdoor have been developed and reported to have good results^1^, ^2^, ^3^. The vascularized fibular graft, introduced in 1979, was designed to restore the blood circulation in cases of ONFH^23^. This procedure requires microvascular surgical techniques.

### The Possible Reasons for the Decline in Osteotomy and Bone Grafting

Both osteotomy and bone grafting as joint‐preserving surgery for ONFH have a long history, and surgeons have worked hard to improve the techniques. Although the number of indications for THA is increasing and joint‐preserving surgery rates are decreasing, for young ONFH patients, THA should not be the easy choice because revision surgery, infection, dislocation, and activity limitations can be lifelong problems. Several factors may be responsible for the decline in these surgical procedures. The characteristics of each joint‐preserving surgery are summarized in Table 4. From the doctor's point of view, the surgery is difficult, and the results are uncertain. For the patient, a long hospital stay is required, followed by long‐term rehabilitation, and it may require removal of the metal implant. In addition, the difficulty of converting to THA as the final treatment option for ONFH may be an important factor. In contrast, if CD‐based regenerative therapy fails, it does not interfere with conversion to THA.

### Limitations of the Study

There are several limitations to this study. First, the US data are national averages based on NIS data and ICD codes, but the Japanese data are biased from 30 high‐volume, teaching‐hospital groups. Second, the third period of US data is of 2 years only. Some other measures could have been taken to get an idea of trends during the period of the Japan data (till 2011). There have been reports on nationwide epidemiology in the United Kingdom^24^, North India, and China^25^. However, only in the US and Japan were we able to find reports on the number or percentage of surgical procedures for ONFH. The US data are considered to be similar to European data. Comprehensive data on surgical treatment for ONFH in other countries are not available. However, in Asia, only Japan is considered to have a bias toward joint preservation in ONFH. Although cell therapy combined with CD is becoming more popular in Europe and the US, its adoption in Japan remains slow, and results are still unknown.

### Conclusions

In conclusion, a comparison of the ONFH procedures in the US and Japan showed that the first option for joint‐preserving surgery was CD in the US, whereas it was osteotomy in Japan, and neither option has been available in the other country.

### Authorship Declaration

All authors listed meet the authorship criteria according to the latest guidelines of the International Committee of Medical Journal Editors, and all authors are in agreement with the manuscript.
